# Genetic interplay between the transcription factors Sp8 and Emx2 in the patterning of the forebrain

**DOI:** 10.1186/1749-8104-2-8

**Published:** 2007-04-30

**Authors:** Andreas Zembrzycki, Gundula Griesel, Anastasia Stoykova, Ahmed Mansouri

**Affiliations:** 1 Max Planck Institute of Biophysical Chemistry, Department of Molecular Cell Biology, Am Fassberg, 37077 Goettingen, Germany; 2 DFG Center for the Molecular Physiology of the Brain, CMPB, Goettingen, Germany; 3 Department of Clinical Neurophysiology, University Goettingen, Robert-Koch-Str., 37075 Goettingen, Germany

## Abstract

**Background:**

The forebrain consists of multiple structures necessary to achieve elaborate functions. Proper patterning is, therefore, a prerequisite for the generation of optimal functional areas. Only a few factors have been shown to control the genetic networks that establish early forebrain patterning.

**Results and conclusion:**

Using conditional inactivation, we show that the transcription factor Sp8 has an essential role in the molecular and functional patterning of the developing telencephalon along the anteroposterior axis by modulating the expression gradients of *Emx2 *and *Pax6*. Moreover, Sp8 is essential for the maintenance of ventral cell identity in the septum and medial ganglionic eminence (MGE). This is probably mediated through a positive regulatory interaction with Fgf8 in the medial wall, and Nkx2.1 in the rostral MGE anlage, and independent of SHH and WNT signaling. Furthermore, *Sp8 *is required during corticogenesis to sustain a normal progenitor pool, and to control preplate splitting, as well as the specification of cellular diversity within distinct cortical layers.

## Background

The mammalian forebrain, with its components the basal ganglia (subpallium) and cortex (pallium), is a result of advanced evolutionary processes. Although several genetic pathways that establish cell diversity within the developing telencephalon have been identified, only a few factors have been shown to control the earliest steps of anteroposterior (A/P) and dorsoventral (D/V) patterning [1].

From embryonic stage E7.5 onwards, the telencephalic vesicles are progressively regionalized through complex interactions of secreted ligands from inductive centers, and by the regionalized or graded expression of transcription factors [1-3]. FGF (Fibroblast Growth Factor) signaling acts downstream of SHH (Sonic Hedgehog) and is required to both specify and promote the proliferation and/or survival of ventral cell types in the telencephalon [4-7], while WNT (Wint) signaling apparently elaborates archicortical morphogenesis [8,9]. Interestingly, modulation of the normal expression gradient of *Fgf8 *in the early cortical primordium alters the molecular location and the size of cortical domains along the A/P axis [10,11]. At the beginning of cortical neurogenesis, several transcription factors display graded expression in cortical progenitors along the main axes, which seems to confer cortical regional specificity [1-3]. The transcription factors Emx2 and Pax6 exhibit an opposing expression gradient along the A/P axis of the forebrain. Accordingly, single mutants of either *Emx2 *or *Pax6 *show a severe shrinkage of the corresponding cortical area, which normally expresses these genes at high levels [2]. Of note, Emx2 is the only factor that has been shown to additionally affect the innervation of thalamic axons into the cortex [1-3].

The zinc-finger transcription factor Sp8 is expressed in the developing nervous system, limbs and the tail bud. Analysis of *Sp8 *knockout mice revealed severe truncations of the limbs and tail, while at the midbrain-hindbrain boundary (MHB) a defect of A/P patterning occurred (12, 13, 14). Interestingly, *Fgf8 *expression is affected in both cases, suggesting that Sp8 may be required for the maintenance of *Fgf8 *activity in these tissues. Recent evidence indicates that abolishment of Sp8 function (in the ventral telencephalon) provokes enhanced apoptosis in progenitors of the dorsal lateral ganglionic eminence (dLGE), causing the loss of specific olfactory bulb interneurons [15]. Additionally, *Sp8 *displays a graded expression pattern in cortical progenitors, with highest expression in the medial pallium.

To gain more insights into the role of Sp8 in the developing telencephalon, we created Foxg1-Cre-mediated conditional *Sp8 *mutants. We present evidence that, in the absence of Sp8, while Shh signaling is normal, the D/V patterning at the medial telencephalic wall is perturbed. Midline derivatives of the subpallium are malformed or completely missing. Additionally, due to the modulation of the graded expression territories of *Emx2 *and *Pax6 *in pallial progenitors, a caudalization of cortical areas occurs. Our study indicates that Sp8 function is required to prevent pallial progenitors from apoptosis, and to control the molecular specification of subsets of cortical layer neurons. This is consistent with an essential role for Sp8 in the patterning of the developing forebrain along the A/P and D/V axes. Furthermore, our findings support the idea of a direct interaction between Sp8 and Emx2 proteins.

## Results

### *Sp8 *mutant brains exhibit multiple malformations

**Figure 1:**
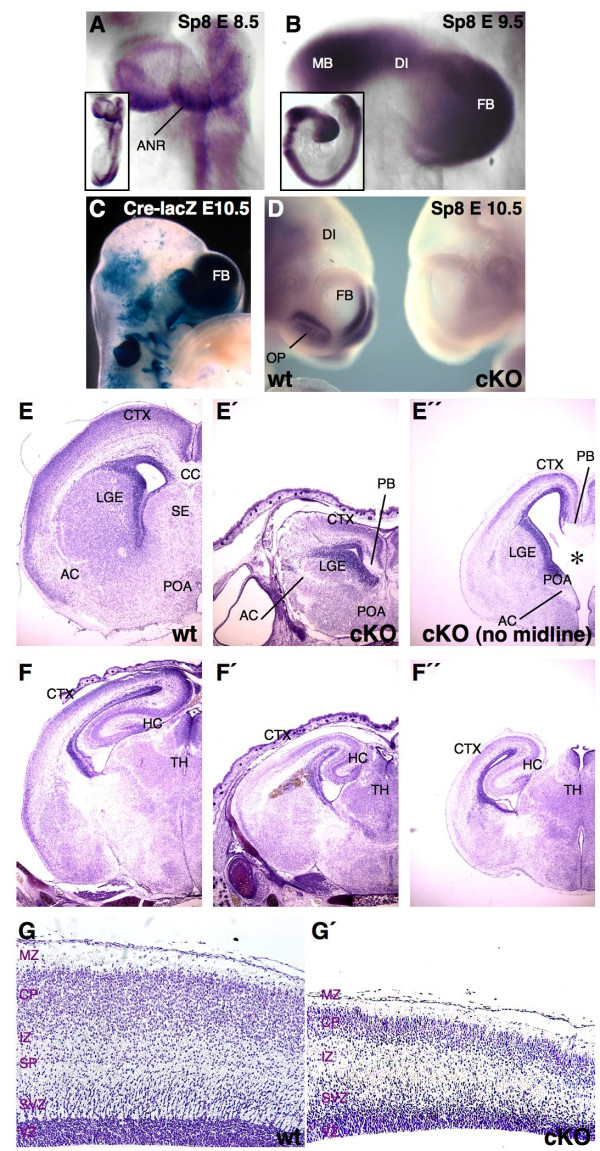
Phenotype of conditional *Sp8 *mutants. **(a, b) **WMISH of *Sp8 *in E8.5 and E9.5 embryos. *Sp8 *is strongly expressed in the anterior neural ridge and in the forebrain neuroepithelium at E8.5 (a). At E9.5, *Sp8 *expression covers the putative forebrain vesicle (b). **(c) **Foxg1-Cre activity, visualized by X-Gal staining, is evident throughout the telencephalon at E10.5. **(d) **Cre recombination ablates *Sp8 *expression in the telencephalon and olfactory placode of cKO at E10.5. **(e, f") **Histological (nissl stained) coronal sections at E18.5. **(e', f') **Mutant brains miss the septum and reveal a reduced size of the telencephalon. **(e', e") **Callosal fibers do not cross the midline and form probst bundles unilaterally. **(g, g') **On (nissl stained) sagittal sections, a strongly reduced cortical diameter is characteristic for cKO at E18.5. With 15% penetrance, cKO brains show an enhanced phenotype, highlighted by the complete absence of midline derivates. These mutants were termed 'cKO no midline' (e", f"). cKO and 'cKO no midline' only differ at rostral levels of the forebrain. In 'cKO no midline' specimens, a delamination of the cortex from the basal telencephalon is apparent medially, as a visible hole (asterisk in e"). Caudally in the brain, the difference between low and high penetrance of the phenotypes is not significant (f', f"). AC, anterior commissure; ANR, anterior neural ridge; CC, corpus callosum; CP, cortical plate; CTX, cortex; DI, diencephalon; FB, forebrain; HC, hippocampus; IZ, intermediate zone; LGE, lateral ganglionic eminence; MB, midbrain; MZ, marginal zone; OP, olfactory placode; PB, probst bundles; POA, preoptic area; SE, septum; SP, subplate; SVZ, subventricular zone; TH, thalamus; VZ, ventricular zone.

At E8.0-E8.5, *Sp8 *is strongly expressed in the anterior neural ridge (Figure 1a). At stage E9.5, *Sp8 *mRNA is apparent in the entire forebrain anlage in a rostral-ventral/high, caudal-dorsal/low gradient. Additionally, *Sp8 *transcripts were detected in the olfactory placode (Figure 1b; data not shown). After E10.5, cortical *Sp8 *mRNA levels decrease, although keeping the typical mediolateral expression gradient in cortical progenitors (Additional data file 1). During later developmental stages, *Sp8 *transcription is further down-regulated within the cortical ventricular zone (VZ), but remains strongly evident throughout adulthood in the septum (SE), dLGE and olfactory system [15] (data not shown). To gain more insights into the role of *Sp8 *during forebrain development, we generated mice with a floxed *Sp8 *locus. *Sp8 *floxed mice were crossed with mice expressing the Cre-recombinase under the control of the *Foxg1 *promoter [16] (Additional data file 1) and the floxed allele was bred to homozygozity (termed cKO).

Homozygous *Sp8 *mutants died at birth. Mutant E10.5 forebrains lacked detectable levels of *Sp8 *mRNA (Figure 1d). At midgestation cKO embryos showed strong craniofacial abnormalities (data not shown). Nissl-stained histological sections revealed that cKO embryos displayed a dysgenesis of the olfactory bulbs (data not shown) and SE, including an almost complete absence of the midline (15% penetrance, n = 25) that resulted in a mild rostral holoprosencephaly (Figure 1e, e', e''; Additional data file 2). In addition, the thickness of the cortical plate of the cKO cortex was reduced (Figure 1g, g'; 63.5% ± 5.1% of control, n = 10). The basal ganglia consisted of a single eminence (Additional data file 2) with a barely discernable constriction between the LGE and MGE. Corticofugal fiber tracts did not cross the midline and instead formed probst bundles (Figure 1e, e', e''). Caudally, neuronal fibers formed bundles between the internal and external capsules (arrows in Figure 1f, f', f'').

### *Sp8 *modulates the D/V patterning of the medial telencephalon

**Figure 2:**
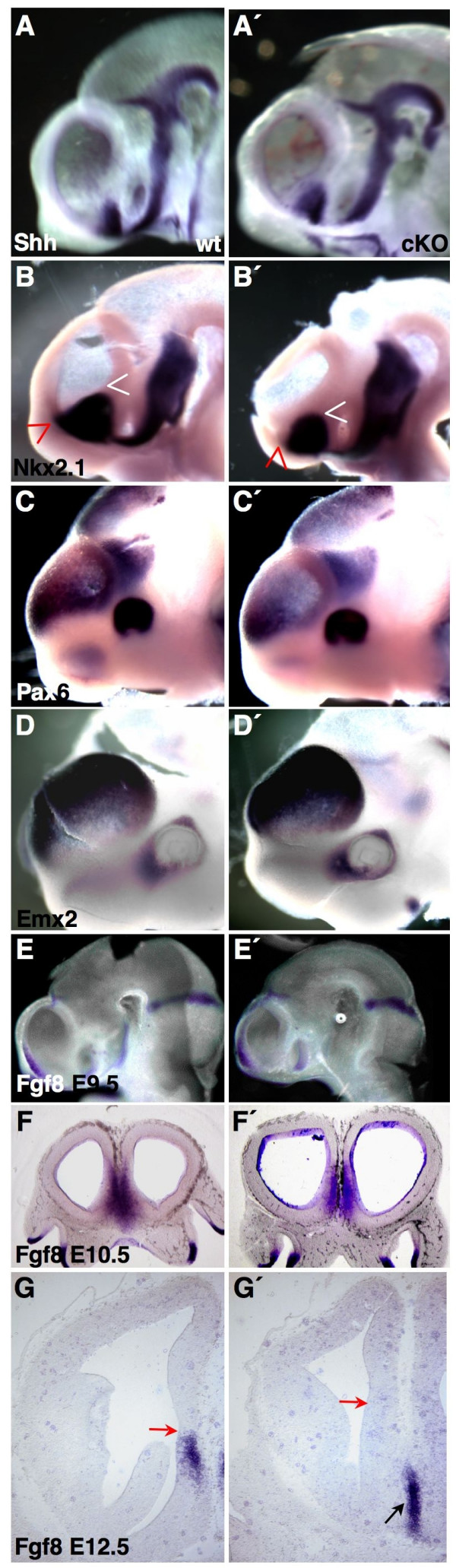
Early patterning of the cKO forebrain. **(a-f') **WMISH and (g, g') ISH on sections. Shh (a, a') and *Fgf8 *expression (e, e') at E9.5 is unchanged in cKO. The septum and large parts of the MGE anlage (red and white arrowheads in (b, b')) are free of *Nkx2.1 *activity. *Pax6 *activity is diminished in cKO (c, c'). *Emx2 *is slightly up-regulated in mutants (d, d'). At E10.5, *Fgf8 *is expressed in the telencephalic midline and septum anlage in both genotypes (f, f'). At E12.5, *Fgf8 *mRNA is specifically lost in the septum (red arrow in (g, g')). The *Fgf8 *positive domain in cKO at E12.5 matches the midline of the POA (black arrow in (g')).

The loss of *Sp8 *activity in the forebrain provokes structural perturbations of the SE and MGE and prompted us to look for molecular markers associated with forebrain patterning. Whole mount *in situ *hybridization (WMISH) at E9.5 (after the initiation of Cre activity [16]) revealed that the mean expression levels of *Pax6 *and *Emx2 *are, conversely, down- and up-regulated in cKO (Figure 2c, c', d, d') compared to wild-type embryos. Importantly, the activity of both *Fgf8 *and *Shh *seemed unchanged in the absence of Sp8 (Figure 2a, a', e, e'). Remarkably, *Nkx2*.*1 *mRNA [17] was reduced in cKO. Rostrally, the septum anlage was free of *Nkx2*.*1 *transcripts (red arrow in Figure 2b, b'), and the caudal portion of the MGE anlage was devoid of *Nkx2.1 *activity (white arrow in Figure 2b, b'). At E10.5, *Fgf8 *mRNA was evident in the telencephalic midline, including the septum anlage (Figure 2f, f'). Interestingly, at E12.5, we found that the *Fgf8 *expression domain in the SE of the mutants was specifically lost (Figure 2g, g'), while the expression of *Shh *[4,7] appeared to be unaffected (data not shown). This suggests that *Sp8 *function might be required for the maintenance of the late expression of *Fgf8 *in the telencephalic midline.

**Figure 3:**
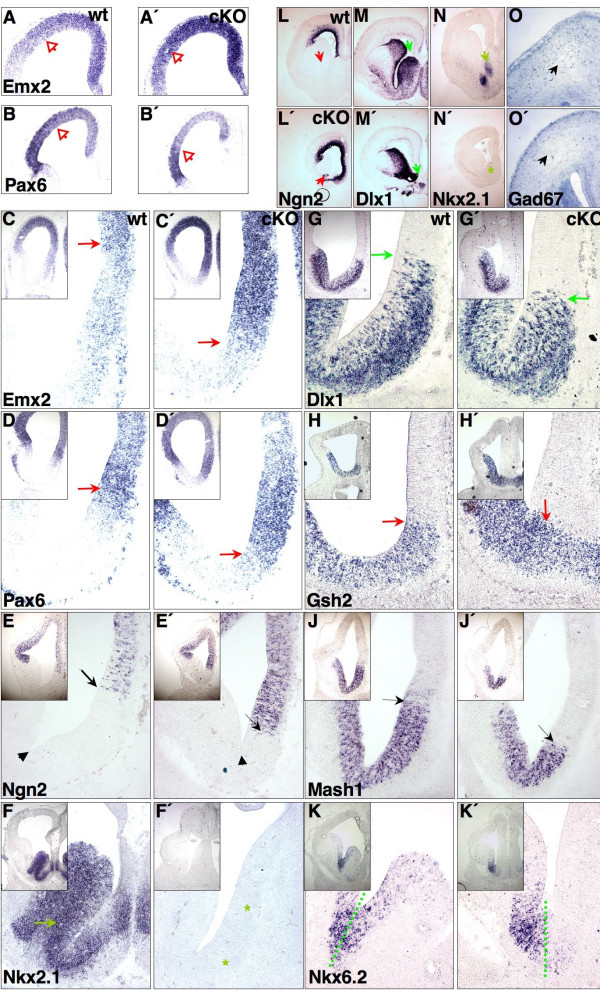
D/V patterning defects at the medial pallial-subpallial boundary (mPSB). ISH on E12.5 (coronal) forebrain sections. Images show the right brain hemisphere. **(c-k') **Blow-up images of the medial/ventral part of each boxed section. In cKO, the pallial markers **(a, a', c, c') ***Emx2*, **(b, b', d, d') ***Pax6*, and **(e, e') ***Ngn2 *expand into the ventral midline. Conversely, ventral markers **(g, g') ***Dlx1*, **(h, h') ***Gsh2*, and **(j, j') ***Mash1 *are not expressed in the dorsal septum. **(f, f') ***Nkx2.1 *activity is lost in the septum and rostral MGE of Sp8 mutants. **(k, k') **The *Nkx6.2+ *domain reflects a rudimentary MGE territory in cKO and contacts the *Emx2*+, *Pax6+ *and *Ngn2+ *midline. Note the **(l, l') **expansion of the *Ngn2+ *domain (arrows) and the **(m, m') **reduction of the *Dlx1+ *domain (arrows) around the midline of the Sp8 deficient telencephalon at E15.5. **(n, n') ***Nkx2.1 *(arrow) remains absent in the septum and rostral MGE of cKO at this stage. **(o, o') **Depletion of (*Gad67*+) interneurons in the Sp8 mutant cerebral cortex (arrow) at E18.5. For a better visualization of the shift in gene expression pattern, the arrowheads in (e, e') point at the constriction between the septum anlage and the LGE. ISH for *Emx2/Pax6 *(a, a', b, b', c, c', d, d') and *Ngn2/Mash1 *riboprobes (e, e', j, j') were performed on adjacent sections.

Next, we examined the patterning of the expression interface at the border of the pallium/subpallium in the medial telencephalic wall, designated medial pallial-subpallial boundary (mPSB). At the mPSB, both the *Emx2+ *and *Pax6+ *expression domains were expanded ventrally (arrows in Figure 3c, c', d, d'). Similarly, the *Pax6 *target gene [18], *Ngn2*, displayed ectopic expression in the presumptive territory of the SE (Figure 3e, e'). In accordance with the reported inhibition of the ventral marker *Mash1 *by Ngn2 [19], the *Mash1+ *territory at the mPSB was reduced in cKO (Figure 3j, j'). A similar down-regulation of the expression of the ventral markers *Gsh2 *[20-22] and *Dlx1 *[23] (arrows in Figure 3h, h', g, g') was also evident in the dorsal SE. At E15.5, the *Ngn2 *expression domain was still massively enlarged in the medial telencephalic wall, and thus, the expression domain of *Dlx1 *remained severely shrunken (Figure 3l, l', m, m'). Intriguingly, despite the partially preserved expression domain of *Nkx2.1 *in the presumptive MGE territory at E9, examination of E12.5 and E15.5 mutant embryos revealed that *Nkx2.1 *expression was almost absent from the subpallium (Figure 3f, f', n, n'). The only exception was a small stripe in the putative caudal ganglionic eminence (data not shown). Furthermore, the *Nkx6.2+ *domain that normally marks the MGE/LGE junction [5] was shifted medially and reached the ventrally expanded *Emx2+*, *Pax6+*, and *Ngn2+ *midline territories in cKO (Figure 3k, k' ; see also Figure 3c, c', d, d', e, e'). Given the loss of substantial parts of the MGE during embryonic development, where the neuronal progenitors mainly generate interneurons [17], cKO cortices contained a strongly reduced amount of *Gad67+ *interneurons at E18.5+ (20.3 ± 2.7% of wild type, n = 3; Figure 3o, o'). Our analysis further indicated that the expression boundaries of *Pax6 *(Figure 3b, b'), *Ngn2 *(Figure 3e, e'), *Dlx1 *(Figure 3g, g'), *Mash1 *(Figure 3j, j'), and *Gsh2 *(Figure 3h, h') at the lateral pallial-subpallial boundary (PSB) were not altered in *Sp8*-deficient brains. This demonstrates that the molecular identity of the lateral pallium and LGE [17,20-22] is not affected by the absence of Sp8. In contrast, our data are consistent with a role for *Sp8 *in the D/V patterning in the medial telencephalic wall.

To study whether the *Sp8 *mutation might affect pattern formation through the Wnt signaling pathway [8], we examined the expression of *Wnt3a*, *Wnt5a *and *Wnt7b *in the cortical hem and the Wnt antagonist *Sfrp2 *in the antihem. No change in the expression of these markers was apparent (data not shown). We concluded that Sp8 might act downstream or independently of Wnt signaling [5,7,10].

### Abnormal cortical arealization and thalamic innervation in *Sp8 *mutants

**Figure 4:**
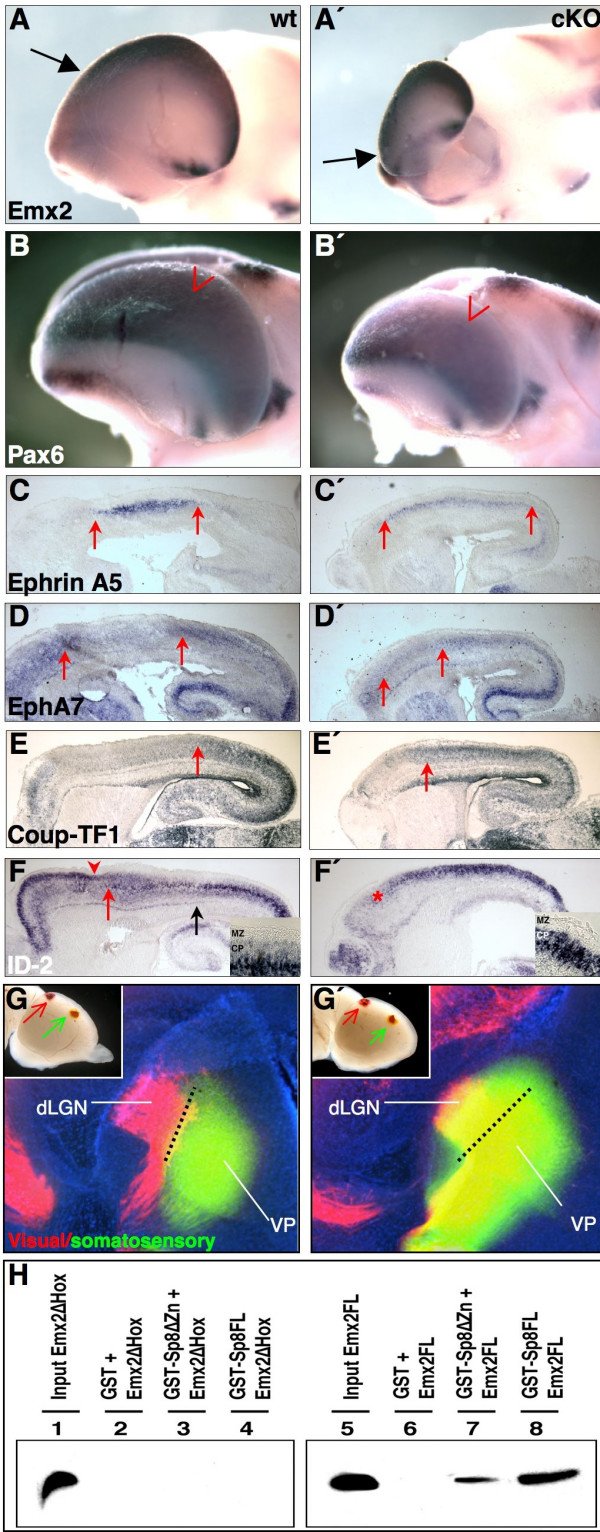
Caudalized gene expression and thalamic innervation in the brain of Sp8 mutants. **(a, a', b, b') **WMISH on dissected E12.5 forebrains, using *Emx2 *and *Pax6 *riboprobes. The *Emx2 *gradient is up-regulated in cKO (arrows in (a, a')). The expression level of *Pax6 *is diminished (arrowheads in (b, b')) in Sp8 mutants. Analysis of the area specific marker genes **(c, c') ***EphrinA5*, **(d, d') ***EphA7*, **(e, e') ***Coup-TF1 *and **(f, f') ***ID-2 *on E18.5 sagittal sections using ISH (rostral is to the left). The visual cortex area in cKO cortices appears expanded towards the rostral brain, as demonstrated by *EphA7*, *ID-2 *and *Coup-TF1 *expression (strong rostral domain of *EphA7 *in (d'), strong *ID-2 *domain in (f'), strong domain of *Coup-TF1 *in (e')). The somatosensory cortex (area between the red arrows in (c, d') and between the black and red arrows in (f)) shifts rostrally in Sp8 conditional mutants (compare (c, d) with (c', d'), and (f) with the area indicated by the asterisk in (f')). The motor cortex expression area appears condensed (compare rostral to left arrow in (c, c', d, d')) in Sp8 deficient specimens. **(g, g') **Coronal sections of E18.5 brains labeled with DiI and DiO and counterstained with DAPI. In controls, DiI, placed in the visual cortex (inset in (g)), retrogradelly labels only cells in the dLGN (g). Placing DiO in the somatosensory cortex (inset in (g)) marks only cells in the VP (g). In the mutants the green dye, placed into somatosensory cortex (inset in (g')) labels cells in the VP and the dLGN (g'). **(h) **GST-pull down reveals that Emx2 lacking the homeobox (Emx2ΔHox) does not bind GST, GST-Sp8 (GST-Sp8FL) or GST-Sp8 lacking zinc fingers (GST-Sp8ΔZn) (lanes 2–4). Full-length Emx2 protein (Emx2FL) does not bind GST, but interacts with GST-Sp8 (GST-Sp8FL) and GST-SP8 lacking zinc fingers (GST-Sp8ΔZn) (lanes 6–8). Lanes 1 and 5 show 10% of the radiolabeled Emx2 isoforms, used as input for the binding assays in lanes 2–4 and 6–8.

In the embryonic brain, *Sp8 *and *Emx2 *show similar expression characteristics along the medial-lateral axis (high at caudomedial levels and low at rostrolateral levels; Figure 3; Additional data file 1). Interestingly, the pallial *Emx2 *gradient is clearly up-regulated in mutant embryos at E12 (arrow in Figures 3a, a' and 4a, a'). Conversely, *Pax6 *normally displays an expression gradient that is high in the rostrolateral and low in the caudomedial pallium, opposing that of *Emx2*. These two genes mutually control their activities in the cortical neuroepithelium [1]. In accordance with the enhanced expression level of *Emx2*, *Pax6 *mRNA is clearly reduced in cKO (arrow in Figure 3b, b' ; Figure 4b, b'). Given the crucial roles played by Emx2 and Pax6 in the arealization of the neocortex [1-3,10,24,25], their altered expression in *Sp8 *mutants prompted us to analyze cortical arealization. The expression of the EphA7 receptor and the EphrinA5 ligand specifically demarcates the regions of the motor/visual and somatosensory cortex, respectively [24,26]. In the cKO, the *EphrinA5 *somatosensory domain extends rostrally (Figure 4c, c'). Accordingly, the motor cortex area, which normally expresses *EphA7 *at high levels, shrinks (Figure 4d, d'). A similar alteration was observed for the characteristic expression of *Coup-TF1*, normally showing a prominent caudal/high to rostral/low expression gradient [21,27]. In *Sp8 *mutants the strong caudal expression domain of *Coup-TF1 *expanded much more rostrally, reaching the presumptive motor cortex (arrows in Figure 4e, e'). A caudalization of the molecular properties along the A/P axis of the cortex was also evident, when *ID-2 *expression was examined. High *ID-2 *levels in upper cortical layers (arrowhead in Figure 4f) of the motor cortex and in layer V in the caudal cortex normally highlight the border between the motor and somatosensory domains [2,3] (red arrow in Figure 4f). In *Sp8 *mutants the caudal *ID-2 *expression territory extends ectopically into the rostral cortex. Additionally, in the presumptive motor cortex, *ID-2 *expression in upper cortical layers was abolished (Figure 4f').

To study whether the observed molecular caudalization of the Sp8cKO cortex reflects alterations of the cortical area identity, we performed retrograde labeling of thalamocortical (TCA) projections by placing crystals of the lypophilic dies DiI (red) and DiO (green) in the presumptive visual cortex or somatosensory area, respectively (insets in Figure 4g, g'). In controls, the red dye was exclusively present in cells within the dorsal lateral geniculate nucleus (dLGE; dorsal from the dashed line in Figure 4g), while the DiO labels (in green) cells in the ventroposterior complex (VP; ventral from the dashed line in Figure 4g). In the Sp8KO brain, the dLGE was labeled by TCA projections coming from both visual and somatosenory cortex (Figure 4g'). These results suggest that the molecular caudalization of the Sp8KO cortex causes a partial change in the cortical area identity (somatosensory to visual fate).

To elucidate whether the regulation of Emx2 by Sp8 might be direct or indirect, we performed biochemical *in vitro *experiments. Glutathione S-transferase (GST)-pull down assays revealed that truncated Emx2 protein, lacking the homeobox, does not interact with GST-Sp8 or GST-Sp8 without zinc fingers (Figure 4h, lanes 3 and 4). However, we found that GST-Sp8 as well as GST-Sp8 lacking zinc fingers are able to bind the full-length Emx2 protein *in vitro *(Figure 4h, lanes 7 and 8). Taken together, this indicates that the *Sp8 *expression gradient in cortical progenitors plays an important role in the correct positioning of distinct cortical domains along the A/P axis of the developing cortex by modulating the expression level of *Emx2*. Furthermore, our findings support the idea of a direct interaction between Sp8 and Emx2 proteins.

### *Sp8 *controls cell survival in the developing forebrain

**Figure 5:**
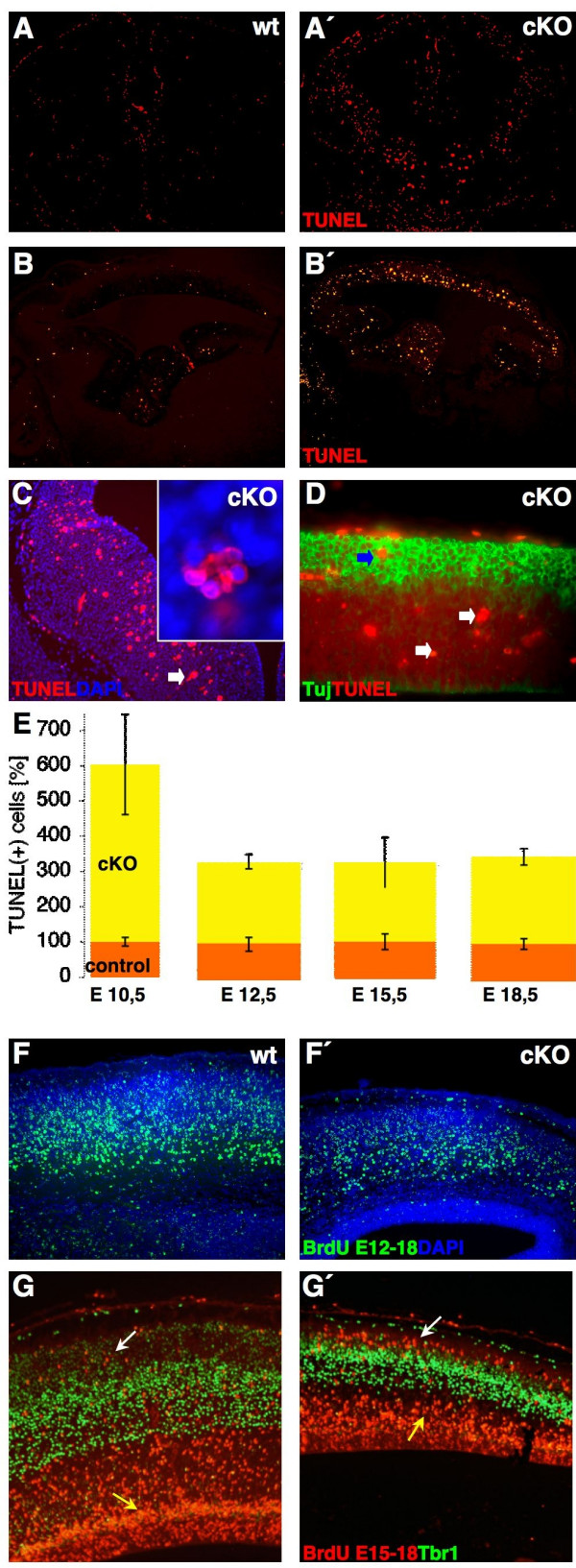
Apoptotic cell death in *Sp8 *deficient brains. TUNEL staining on **(a, a', c) **E12.5 (coronal) and **(b, b', d) **E15.5 forebrain sections (sagittal; rostral is to the left). The arrow in (c) demarcates a typical cluster of apoptotic cells, found in cKO, and found from E12.5 to E18.5. Clusters typically contained five to seven apoptotic cells (blow-up in (c)). (d) Tuj/TUNEL double staining in cKO reveals TUNEL+ cells within the putative CP (blue arrow) and presumptive proliferative zones and the IZ (white arrows). **(e) **Quantification of TUNEL+ nuclei on E10.5, E12.5, E15.5 and E18.5 forebrain sections (n = 3–4 for each stage) shows an increased cell death without Sp8 function. Fate mapping of **(f, f') **early-born and **(g, g') **late-born pallial neurons (using BrdU injection at E12.5 or E15.5) on E18.5 sagittal sections. Putative early-born neurons migrate through the CP (f, f'). Their number is diminished in mutant cortices (f') compared to controls (f). In cKO, BrdU+ neurons populate ectopic positions in the upper CP, when compared to the relative position of Tbr1 immunoreactive cells (white arrow in (g')), possibly reflecting the thinned cortex. BrdU labeling at E15.5 appears reduced in the SVZ of mutants (yellow arrow in (g')).

The hypoplasia provoked in the forebrain of cKO led us to speculate that this may be related to defects in cell proliferation and/or apoptosis [28,29]. Such alterations were additionally observed in *Foxg1 *knockout mice [30]. However, *Foxg1 *activity was not affected in *Sp8 *mutants (data not shown). We therefore determined the bromodeoxyuridine (BrdU) labeling index (BrdU pulse), M-Phase index (pH3 staining), and cell cycle exit [30,31] (BrdU/iododeoxyuridine (IdU) double labeling) parameters in the forebrain of cKO at E12.5. None of these parameters were significantly altered in mutant forebrains (Additional data file 3). Furthermore, neither ectopic mitosis nor premature differentiation seems to occur in cKO (Additional data file 3). However, using a TUNEL assay at E12.5, we detected a dramatic increase of apoptosis in forebrain sections (Figure 5a, a'). TUNEL+ cells were found randomly distributed in the dorsal and basal telencephalon (Figure 5a, a', b, b' ; data not shown). The apoptotic cells formed clusters consisting of three to six individual nuclei (arrow in Figure 5c). Counting of TUNEL+ cells on E10.5, E15.5 and E18.5 sections revealed that the *Sp8*-deficient forebrains contained six times more apoptotic cells at E10.5 and three times more from E12.5 to E18.5 compared to controls (Figure 5e). TUNEL+ nuclei were found in putative proliferative regions (white arrows in Figure 5d) and in the cortical plate proper (CP; blue arrow in Figure 5d). *Sp8 *deficiency possibly affects the survival of some post-mitotic neurons (Figure 5d; 78.4% TUNEL+/Tuj- at E15.5, n = 2), but mainly early (E10.5-E12.5) neuronal progenitors. Thus, the forebrain hypoplasia in cKO appears as a consequence of the apoptosis-induced loss of progenitors.

We next wanted to know whether the observed loss of progenitors might affect the generation of specifically early- or late-born cortical neurons [32]. By injecting BrdU at E12.5 or E15.5 and sampling at E18.5, we created specimens that had BrdU exclusively incorporated in early- (E12.5) or late-born (E15.5) neurons. Analysis of the samples injected at E12.5 identified cells populating deep and intermediate positions of the CP (Figure 5f). In accordance with the detected apoptosis, the number of BrdU+ nuclei in mutant brains was reduced (Figure 5f' ; 64.7 ± 7.8% of wild type, n = 3), pointing to a diminished progenitor pool. Cells labeled with BrdU at E15.5 populated mainly deep compartments (VZ, subventricular zone (SVZ), intermediate zone (IZ)) of the cortex of both genotypes (Figure 5g, g'). However, the amount of BrdU+ cells within the putative SVZ, which were recently shown to generate exclusively upper cortical layers [33-35], appeared reduced in the mutants (yellow arrow in Figure 5g'). Conversely, more BrdU-labeled cells were detected in superficial positions in the CP (white arrow in Figure 5g'), most probably reflecting the reduction in the distance from the ventricular to the marginal zone in the mutant cortex.

### The loss of *Sp8 *results in defective preplate splitting

**Figure 6:**
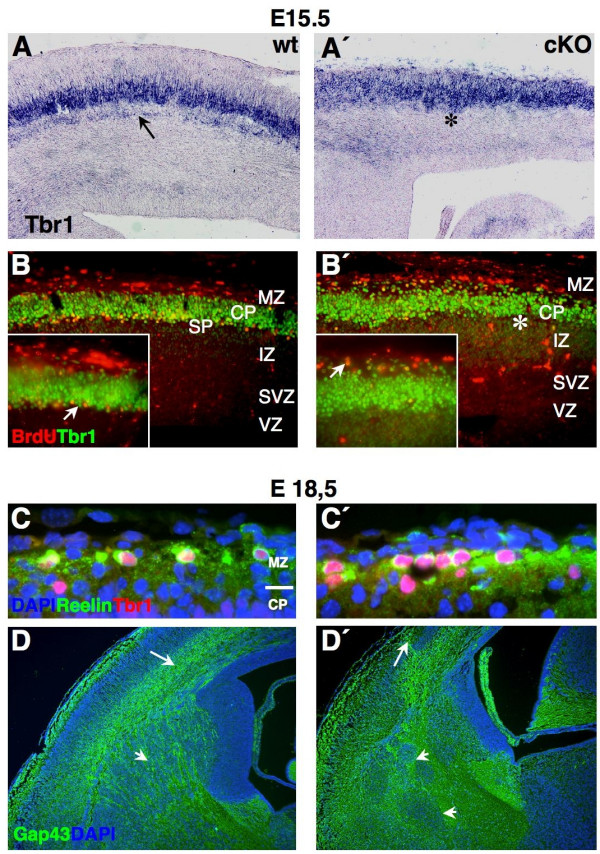
Defective preplate splitting and subplate development. **(a, a') **ISH for *Tbr1 *transcript on E15.5 coronal sections (medial is to the right). **(b, b') **Immunohistochemical co-detection of Tbr1 and BrdU on E15.5 sagittal sections (rostral is to the left). Mutants lack well separated/positioned *Tbr1*+, putative subplate cells at E15.5 (arrow and asterisk in (a, a')). At this stage, BrdU+ (injected E11) cells co-labeled with Tbr1 form a superplate-like structure by populating the upper CP/MZ in cKO (arrow in (b)). In controls, those cells settle in the subplate area (arrow in (b)). Arrows indicate the dislocation of BrdU+/*Tbr1*+ cells in mutants (b'). Co-detection of Reelin- and Tbr1 protein in the MZ on coronal sections at E18.5 (c, c') (medial is to the right) cKO showed more Reelin+/Tbr1+ (putative Cajal-Retzius cells) in the MZ (c'). The PPL does not split properly in cKO; additionally Gap43+ axons (assayed on E18.5 coronal sections) form bundles around the internal capsule (arrowheads in (d, d') and some project ectopically into the MZ (arrow in (d, d')).

The apoptotic cell death detected in the forebrain of *Sp8 *mutant embryos may also affect neurogenesis and early cortical layer development. The cortical preplate (PPL) consists of early-born (E10-E11.5) neurons of pallial origin. Later-born neurons will progressively split this domain into the marginal zone (MZ), CP and subplate (SP) [36,37]. We used the *Tbr1 *riboprobe to label early PPL and SP populations [38,39] at mid-gestation. While in controls *Tbr1+ *SP cells are well separated from *Tbr1+ *CP cells (Figure 6a), this does not occur in cKO embryos (Figure 6a').

Furthermore, we used a cell labeling approach consisting of injecting BrdU at E11 to label SP cells [36,40] and then harvesting tissue after a visible separation of SP and CP at E15 (Figure 6a). In addition, the co-detection of BrdU and Tbr1 enabled us to follow the laminar position of the double positive cells, which in controls were found in the SP (arrow in Figure 6b). In contrast, in cKO these cells were located in virtually the most superficial part of the cortical plate (compare Figure 6b, b'). We conclude that proper PPL splitting does not occur in Sp8cKO.

In accordance with defective SP formation [37-40], Gap43 antibody staining revealed that the subcortical connectivity and the axonal wiring appeared abnormal in mutants. In contrast to controls, Gap43+ fibers in *Sp8 *mutants formed aberrant bundles within the internal capsule, with some axons projecting ectopically towards the MZ (arrows and arrowheads in Figure 6d, d') and basal telencephalon (Additional data file 2 (d, d'')).

### Perturbed specification of distinct cortical layer neurons in *Sp8 *mutants

**Figure 7:**
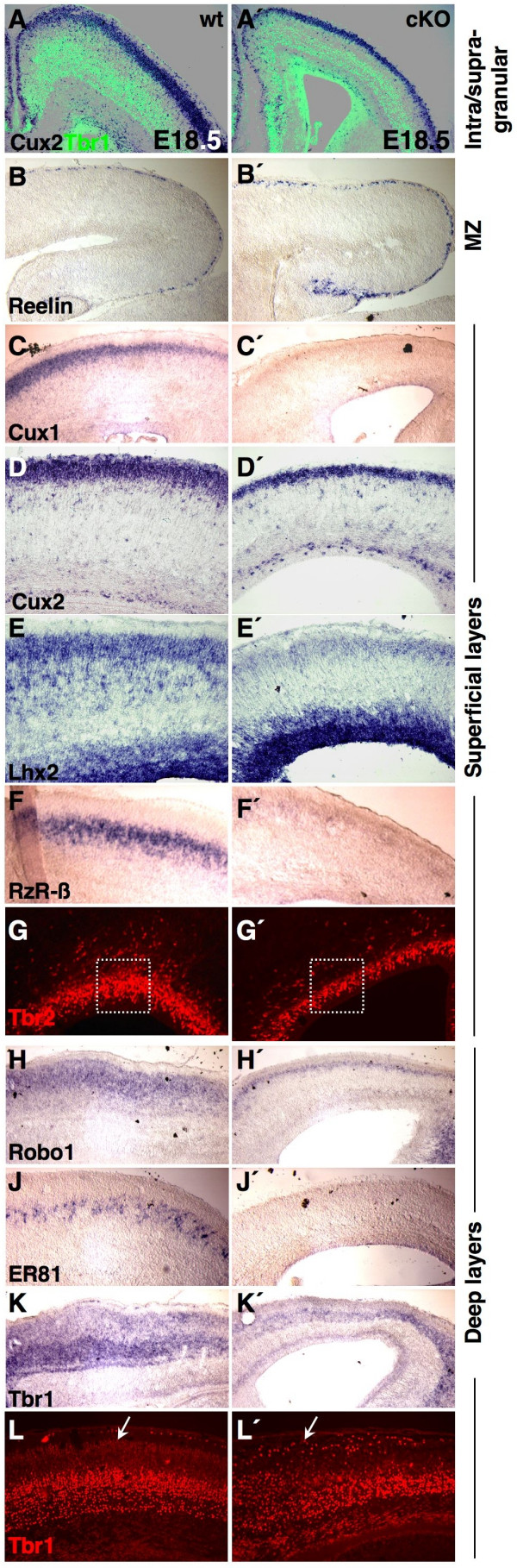
Specification of individual cortical layer neurons. Coronal (E18.5) sections were double labeled with the *Cux2 *riboprobe and the Tbr1 antibody. **(a, a') ***Cux2+ *and *Tbr1*+ cell populations are separated from each other, and the generation of intra-/supragranular layers seems preserved in Sp8 mutants. ISH of layer specific marker genes on **(c, c', f, f', j, j') **E18.5 coronal (medial is to the right) and **(b, b', d, d', e, e', h, h', k, k') **sagittal (rostral is to the left) forebrain sections. **(g, g', l, l') **Detection of Tbr1/2 protein on E18.5 coronal sections (medial is to the right). A reduced expression of *Cux2 *(d, d'), *Lhx2 *(e, e'), *Robo1 *(h, h') and *Tbr1 *(k, k') is visible in mutant cortices. In Sp8cKO, the subpopulations of *Cux1+ *(c, c'), *RzR-β+ *(f, f'), and *ER81+ *(j, j') cortical neurons are not molecularly specified. Tbr2 immunoreactive progenitors are diminished in the proliferative compartment of the Sp8 mutant cortex (box in (g, g')). Immunohistochemistry reveals that some *Tbr1*+ cells ectopically populated the upper CP and MZ in Sp8 mutants (arrow in (l')).

Because the detected PPL defect may provoke additional abnormalities, we investigated whether the generation of infra-/supragranular layers and the differentiation of specific neuronal layers were altered in cKO. We therefore labeled late-born/upper cortical neurons with the *Cux2 *riboprobe [34,35], combined with immunolabeling for Tbr1, tracing early-born/deep layer neurons [41]. We could not observe co-localization (Figure 7a, a') of both markers, demonstrating that the basic laminar organization of the cortex and the switch from early to late neuronal fate is not altered in mutants. To examine whether *Sp8 *might be required for the specification of distinct cortical neuron subtypes, we assayed layer-specific marker genes at E18.5. Recent findings [36,41] indicate that *Tbr1 *activity is able to promote the specification of the SP and layer VI. In accordance with defective preplate splitting and the apoptotic cell death of progenitors in cKO, the analysis of Tbr1 expression by immunohistochemistry or *in situ *hybridization revealed ectopic *Tbr1*+ cells in the uppermost region of the CP (arrows in Figure 7l'), and a less thick band corresponding to layer VI of the deep CP (Figure 7k, k'). In addition, *ER81 *was used to trace a subpopulation of layer V neurons [42], but we could not detect *ER81 *mRNA in CP neurons at E18.5 (Figure 7j, j'). However, the expression of another marker of layer V neurons (*Robo1 *[42]) appeared to be only diminished in cKO (Figure 7h, h'), suggesting that only a subset of lower cortical layers might not be correctly specified.

The orphan nuclear receptor *RzR-β *was utilized to follow layer IV genesis [42]. At E18.5, *RzR-β *transcripts were completely missing in the CP of mutants (compare Figure 7f, f'). Moreover, the expression of Cux proteins in SVZ progenitors was recently shown to promote the fate specification of late-born neurons [34,35]. Accordingly, we found that in the mutant, although *Cux2 *expression was reduced (Figure 7d, d'), *Cux1 *mRNA could not be detected at E18.5 (Figure 7c, c'). Along the same line of evidence, the expression of an additional upper layer neuron marker, *Lhx2*, is also highly down-regulated in the cortical plate of *Sp8 *mutants (Figure 7e, e'). This suggests that a reduction in the generation of late-born/upper cortical layer neurons occurs. We assayed Tbr2 immmunoreactivity. Tbr2 is a specific marker for basal/SVZ progenitors [43], which predominantly generate the upper cortical layers [34,35]. In *Sp8 *mutants, the population of Tbr2+ (basal) progenitors was significantly reduced at E18.5 (49.4 ± 4.3% of controls, n = 3; Figure 7g, g'). This is consistent with a diminished pool of late progenitors, resulting in a diminished generation of upper cortical layer neurons in conditional *Sp8 *mutants.

The MZ mostly consists of Reelin+ Cajal-Retzius cells [1,9,37]. Using *in situ *hybridization (ISH) for *Reelin *mRNA (and a Reelin antibody for quantification) we found more *Reelin+ *neurons in cKO than in control littermates (142.2 ± 6.4% of controls; Figures 6c, c' and 7b, b'). In summary, these findings support the idea that the lack of *Sp8 *function during early neurogenesis is responsible for a severe depletion of the early and the late cortical progenitor pool, resulting in a misspecification of distinct cortical neuron subtypes, such as Cux1+, Lhx2+, RzR-β+, and ER81+ lineages.

## Discussion

We used conditional inactivation to study the role of *Sp8*, the ortholog of the *Drosophila *transcription factor buttonhead [13], during murine forebrain development. We report that the absence of Sp8 provokes a morphological dysplasia of the rostromedial forebrain, perturbs A/P patterning and enhances apoptosis of neuronal progenitors. A marker analysis further revealed that although the layering of the mutant cerebral cortex seems normal, Sp8 function is required for the specification of neuronal subpopulations.

### *Sp8 *has an essential role in the formation of the telencephalic midline

One morphologically apparent defect was the dysgenesis of the septum. On the molecular level, this might result from the ventral expansion of *Emx2*, *Pax6 *and *Ngn2 *expression territories. As a consequence, the expression of several ventral markers, such as *Fgf8*, *Mash1*, *Dlx1*, and most importantly, *Nkx2.1 *is regionally down-regulated or completely abolished. Interestingly, *Shh *and *Wnt *expression seems to be preserved in the mutant, suggesting that the observed perturbation is independent of these signaling pathways. Our findings suggest that *Sp8 *might have a critical role in the maintenance of gene activity at the mPSB, since early marker expression is not affected. Interestingly, both *Sp8 *and *Fgf8 *are expressed in the septum anlage at early developmental stages. Recent findings demonstrate that FGF signaling is acting downstream of Shh to propagate ventral telencephalic cell types and to promote their survival [6]. Our study reveals that, while preserved in the midline and septum anlage until E10.5, the expression of *Fgf8 *(in the septum) and *Nkx2.1 *(in the septum and rostral MGE) is completely abolished at E12.5. Moreover, a recent report provides evidence that Fgf8 may regulate *Nkx2.1 *expression, interfering with the axial patterning of the telencephalon [7], therefore suggesting that the midline defect in cKO could be mediated through Fgf8.

**Figure 8:**
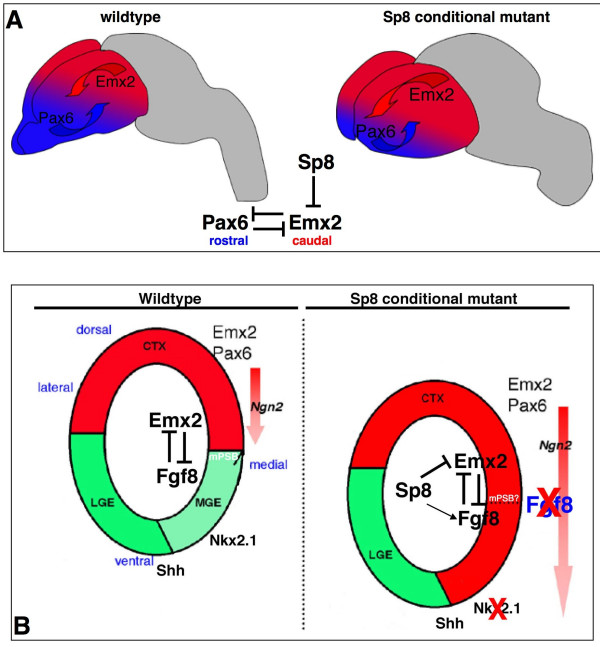
Role of *Sp8 *in the forebrain. **(a) **A scheme illustrating the genetic interplay between Sp8 and Emx2. Emx2 and Pax6 mutually affect their graded expression characteristics along the A/P axis (red and blue arrows). Sp8 acts as a repressor of Emx2 to pattern the forebrain along the A/P axis. In cKO, the mutant *Emx2 *expression domain (red) is expanded rostrally. Accordingly, *Pax6 *expression territory (blue) is diminished. **(b) **Scheme recapitulating the gene expression of marker genes at the medial and lateral PSB. In conditional Sp8 mutant, a dorsalization of gene activity around the midline occurs, as highlighted by the ventral expansion of the domain marked in red. Dorsal: red domain, *Pax6*, *Emx2*, *Ngn2*; ventral: green and light-green domain, *Gsh2*, *Dlx1*, *Mash1*. Reciprocally, the expression area of ventral markers is lost in the dorsal septum (light green domain). Accordingly, *Fgf8 *and *Nkx2.1 *expression domains are not maintained in the mutant cortical midline. We favor a model in which Sp8 regulates the AP regionalization of cortex by directly suppressing Emx2, while the role of Sp8 in the maintenance of Nkx2.1 activity in SE and MGE could consist of controlling Fgf8.

It was recently found that *Sp8 *is down-regulated in the medial telencephalon of *Fgf8 *hypomorphic and conditional mutants [7]. Strikingly, it was demonstrated by three independent studies that Sp8 might positively regulate *Fgf8 *expression in the mouse AER [12,13], and in the zebrafish pectoral fin [44]. Conversely, the loss of *Sp8 *in the MHB provokes an expansion of the *Fgf8 *expression domain [14]. Therefore, an attractive interpretation of the defect at the medial wall might be that a context-dependent bidirectional interaction between *Fgf8 *and *Sp8 *is responsible for the correct patterning of the ventro-medial telencephalon. Recently, FGF signaling was shown to be crucial for the genesis of the SE [45]. In the absence of late *Fgf8 *activity in Sp8cKO, the septal territory abnormally expresses dorsal markers, and therefore, might acquire pallial properties (Figure 8), supporting the idea that Fgf8 and Sp8 may also be required for sustaining ventral cellular identity of septal precursors.

Although *Sp8 *is expressed in the dLGE, the patterning at the PSB is not disturbed. Two possibilities might be envisioned: first, the D/V patterning at the PSB is established before E9, 5; and second, functional redundancy may exist between *Sp8 *and the closely related transcription factor *Sp9 *[44] in the ventral telencephalon (Additional data file 1).

### *Sp8 *affects cortical arealization along the A/P axis

We recently have shown that *Sp8 *knockout mice display a patterning defect at the MHB [14]. In the present study we demonstrate that, in addition to its role in the formation of a normal mPSB, Sp8 is necessary for the molecular arealization of the cerebral cortex along the A/P axis. The arealization of the early cortical primordium is dependent on the regionalized expression of ligands belonging to the FGF, WNT/BMP and epidermal growth factor signaling pathways, produced by the anterior neural ridge (ANR), cortical hem, roof plate and antihem, respectively [1,3]. Such ligands are assumed to control the graded expression of transcription factors, encoding positional pattern, and specific for distinct cortical fields. So far, only a few regionally enriched transcription factors have been shown to be critical for this process. For instance, in mice where either *Pax6 *or *Emx2 *is not functional, the corresponding rostral and caudal cortical regions, where these genes display highest expression, appear malformed and cortical areas are displaced in opposite directions [2,46].

Our findings suggest that the inactivation of *Sp8 *in the forebrain causes a prominent caudalization of the molecular properties of the cortical neuroepithelium, as highlighted by the ectopic rostral expansion of the expression domains of the regionally enriched marker genes *Coup-TF1*, *EphrinA5 *and *EphA7*. Furthermore, Sp8cKO cortices show an enhanced *Emx2 *and a reciprocally down-regulated *Pax6 *expression gradient. This supports the notion that the genetic interplay between Pax6 and Emx2 is controlling the establishment of their normal expression gradients [47]. However, the loss of *Emx2 *or *Pax6 *function does not affect the expression of *Sp8*, suggesting that *Sp8 *acts upstream of these genes (Additional data file 1).

FGF signaling from the ANR plays a crucial role in the patterning along the cortical A/P axis [1]. Evidence has been presented that *Emx2 *might indirectly control cortical arealization, through the regulation of *Fgf8 *[10]. Recent data, however, challenged such a view by demonstrating that *Emx2 *may operate directly, and independent of, *Fgf8 *to specify cortical areas [25]. In addition, it was shown that thalamocortical connectivity is affected only in the absence of proper *Emx2 *function [48], and conversely does not change in *Fgf8 *hypomorphic cortices *in vivo *[11]. In good agreement with these findings we observe defects in thalamocortical projections in Sp8cKO cortices. Notably, the early FGF signaling from the ANR does not seem to be affected in the Sp8cKO forebrain until at least E10.5. Taken together, our findings strongly suggest leading roles for *Emx2 *and *Sp8 *in cortical arealization.

In agreement with findings in *Drosophila*, we show a direct interaction between Sp8 and Emx2 proteins *in vitro*, indicating a conservation of this regulatory pathway [49].

### *Sp8 *plays a critical role in cortical neurogenesis

Enhanced apoptosis was detected in the limbs [13] and basal telencephalon in Sp8 loss-of-function mice [15]. We show here that the forebrain hypoplasia in Sp8cKO mice is primarily due to cell death, affecting both early and late progenitor pools of dorsal and ventral telencephalon.

We further found that when the function of Sp8 is abolished, the preplate splitting is defective, and the MZ contains more Reelin+ cells [50], possibly due to the enhanced Emx2 expression in the mutant cortex. The basic lamination of the cortex is not compromised in cKO. However, the specification of particular neuronal subtypes (such as *ER81-*, *RzR-β-*, and *Cux1*-positive) is affected. Similarly, conditional ablation of *Sp8 *in the basal telencephalon results in misspecification of a subset of interneurons [15].

## Conclusion

Our findings provide evidence that the transcription factor Sp8 is required for both the early patterning of the forebrain and the specification and survival of ventral cell types of the telencephalon. Our findings support the idea that a direct interaction between Sp8 and Emx2 controls D/V patterning of the medial telencephalon, functional arealization along the A/P axis, and the specification of subpopulations of cortical layers.

## Materials and methods

### Generation of *Sp8 *conditional mutant mice

Animal treatment and housing was in agreement with the regulations of LAVES (Landesamt für Verbraucherschutz und Lebensmittelsicherheit) in Oldenburg. The different alleles of *Sp8 *are represented in Additional data file 1. Exons are indicated by black boxes, LoxP sites by black triangles and FRT (Flip recombination target) sites by white triangles. The LoxP-FRT-PGKneo-FRT cassette was inserted 5' to exon 1 and the second LoxP site 3' to exon 3. Southern blot analysis, using probes A, B and C (Figure 1b) identified recombinant clones. Hybridization with probe D (Additional data file 1) confirmed the deletion of the wild-type *Sp8 *allele. Homozygous *Sp8 *floxed mice were maintained on a C57/BL6 background. Conditional Sp8 knockout animals were generated by mating *Sp8 *floxed mice with Foxg1-Cre mice [16] (cKO). Cre activity was monitored using R26R reporter mice [51]. Cre+ cells were traced in triple transgenic mice, obtained by crossing *Sp8 *floxed heterozygous mice (positive for the Cre recombinase) with R26R mice (cKO-R26R).

Genotyping was done by PCR using the following primers: Sp8 (1: CCA-ATG-GGA-GGA-AAA-CAC-ACC-CCC-TCT-TAC-TCC-TC, 2: CCA-GCT-TCC-TGG-ACT-CTT-TCA-GTA-TAG-TTT-TGA-AG, 3: GCG-TGC-AAT-CCA-TCT-TGT-TCA-ATG-GCC-GAT-C); Cre (creF: ATG-CTT-CTG-TCC-GTT-TGC-CG, creR: CCT-GTT-TTG-CAC-GTT-CAC-CG); β-galactosidase (lacZF: TTG-GCG-TAA-GTG-AAG-CGA-C, lacZR: AGC-GGC-TGA-TGT-TGA-ACT-G).

### Embryo recovery and tissue sampling

Pregnancy of mated mice was determined by the appearance of the vaginal plug and defined as day E0.5. Staging of embryos was done according to the plug date. Mice were killed by cervical dislocation. Embryos or tissues were dissected, washed in cold phosphate-buffered saline (PBS) and fixed in 4% PFA/PBS (paraformaldehyde) for several hours overnight. After rinsing in PBS, tissues were processed for standard paraffin- or cryo-embedding. Tissues were cryo-protected by overnight incubation in 30% sucrose/PBS at 4°C. Embedding was done in tissue tec (Jung, Nussloch, Germany) and freezing on dry ice. For whole mount ISH, dissected embryos were processed through a methanol series and kept at -20°C.

### Immunohistochemistry, X-Gal staining and *in situ *hybridization

Immunohistochemistry was performed on 18 μm cryosections, or paraffin embedded sections of 5 μm to 10 μm thickness. Antigens were generally unmasked by boiling in citrate buffer (Vector, Burlingame, CA, USA), as described elsewhere. Primary antibodies were μ-Pax6 (Babco, Richmond, CA, USA), μ-Gap43, μ-Tuj (Covance, Berkeley, CA, USA), μ-BrdU, μ-phospho-HistoneH3 (Abcam, Cambridge, UK), μ-BrdU/IdU (Caltag, Burlingame, CA, USA), μ-Tbr1+2 (gift from R Hevner), and μ-Reelin (gift from A Goffinet). Paraffin sections were dewaxed, rehydrated and rinsed in PBS. Cryosections were washed in PBS and postfixed in 4% PFA/PBS. After unmasking, sections were blocked in a solution containing PBT (PBS + 0.1% TritonX) and 10% FCS (fetal calf serum) for 30 minutes. Primary antibodies were incubated overnight at 4°C in blocking solution. Secondary antibodies were diluted 1:500 in blocking solution and incubated for 1–2 hours at room temperature. Secondary antibodies were Alexa594- or Alexa488-conjugated and raised against mouse-, rabbit- or rat antigens (Molecular Probes, Karlsruhe, Germany). Before mounting, sections were rinsed three times in PBT and sealed with Vectashield mounting medium, containing DAPI as nuclear counterstain (Vector).

X-Gal staining was performed on whole mount tissue or 18 μm cryosections. β-Galactosidase activity was developed in staining solution (PBS, 1 mg/ml X-Gal, 2 mM MgCl_2_, 0.01% SDS, 0.02% NP40, 5 mM K_3_Fe(CN)_6_, 5 mM K_4_Fe(CN)_6_) for several hours overnight at 37°C. Specimens were then washed in PBS and postfixed in PFA. Sections were counterstained in a solution containing 0.1% neutral red.

ISH on 12 μm to 18 μm cryosections and whole mount ISH were performed using digoxygenin labeled riboprobes, as described previously [13,14]. Histological analysis was done on nissl stained or neutral red counterstained sections, following standard procedures.

### Axon tracing

E18.5 brains were fixed in PFA overnight and dissected from the skull. After hemi-sectioning the brains with a blade, crystals of DiI and DiO (Molecular Probes) were placed into multiple, but comparable, locations of the cortex [11,48] across genotypes (n = 6 hemi-sections per genotype and experiment). The diffusion of the tracers was allowed to proceed for 4 weeks in PFA at 37°C. After embedding the brains in 5% LMP-Agarose, 100 μm coronal sections were cut on a vibratome and counterstained with DAPI containing mounting medium (Vector).

### BrdU labeling and TUNEL assay

BrdU and IdU (Sigma, Seelze, Germany) uptake experiments were done by intraperitoneal injection (50 μg/g body weight) of nucleotides into pregnant mice. For pulse labeling, injected mice were sacrificed 30 minutes after injection. Tissues of BrdU-injected embryos were processed for paraffin embedding. Subsequently, 5–10 μm sections were used for immunohistochemistry.

The S-Phase labeling index was estimated by dividing DAPI+ from BrdU+ cells on complete forebrain sections. For fate mapping purposes, BrdU was injected at varying time points from E10.5 to E15.5. Tissues were dissected on E15.5 to E18.5, respectively. The cell cycle length was estimated by sequential BrdU/IdU injection, according to [30,31]. The (cell cycle) leaving fraction was counted by dividing IdU+/BrdU+ cells from IdU+ only cells on forebrain sections.

TUNEL assay was performed on 5 μm paraffin sections of E10, E12, E15 and E18 brains using a ApopTag Red In Situ Apoptosis Detection kit (Chemicon, Hampshire, UK) and following the manufacturer's advice. The amount of TUNEL+ cells in control specimens of every stage was defined as 100%. Apoptotic cell clusters were identified in cKO from E12.5 to E18.5 and counted as one apoptotic cell.

### *In vitro *pull-down assay

The cDNA encoding Sp8 (Sp8FL, AA 1–486) and Sp8 lacking zinc fingers (Sp8ΔZn, AA 1–355) were amplified by PCR (MGI-Clone 2443471) using the following PCR primers, adding 5' *Bam*HI and 3' *Eco*RI restriction sites: Sp8FL forward and Sp8ΔZn forward, G CGC GGA TCC ATG CTT GCT GCT ACC TGT AAT AAG ATC; Sp8FL reverse, G CGC GAA TTC CTC CAG GCC GTT GCG GTG; Sp8ΔZn reverse, G CGC GAA TTC CAG CCC TTT GCG ACG CAG GC. PCR products were then subcloned in frame into the *Bam*HI and *Eco*RI restriction sites of the pGEX-4T-3 expression vector (Promega, Madison, WI, USA). GST and GST-Sp8 fusion proteins were expressed in *Escherichia coli *and purified following standard protocols. Equal amounts of GST-Sp8 proteins or GST were incubated with gluthathione-sepharose beads (Amersham, Piscataway, NJ, USA) and washed in PBS.

The cDNA encoding Emx2 (Emx2FL, AA 1–253) and Emx2 lacking the homeobox (Emx2ΔHox, AA 1–144) were amplified by PCR using the following PCR primers, adding 5' *Eco*RI and 3' *Not*I restriction sites: Emx2FL forward and Emx2ΔHox forward, G CGC GAA TTC ATG TTT CAG CCG GCG CC; Emx2FL reverse, GCG CGC GGC CGC ATC GTC TGA GGT CAC ATC; Emx2ΔHox reverse, GCG CGC GGC CGC GCC AGG GGT AGA AGG TGG ACG. Template DNA containing the Emx2 open reading frame was kindly provided by A Mallamaci. Amplified PCR products were then subcloned into the *Eco*RI and *Not*I restriction sites of the pCMV-TNT vector (Promega). [^35^S]-methionine labeling of Emx2 proteins was performed using the TNT Quick Coupled Transcription/Translation system (Promega).

The GST and GST-Sp8 bound beads were then incubated with [^35^S]-methionine-labeled Emx2 protein isoforms in 0.4 ml binding buffer (0.1 M NaCl, 0.01% NP-40). After incubation for 2 hours at 4°C, beads were washed five times in 400 μl binding buffer and then boiled in 40 μl of 10 × SDS-PAGE loading buffer (10% SDS, 10 mM β-mercaptoethanol, 20% glycerol, 0.2 M Tris-HCl, pH 6.8, 0.05% bromophenolblue). Solubilized proteins were separated by 15% SDS-PAGE and the radiolabeled proteins visualized by autoradiography.

### Statistics and data processing

Statistics were calculated with Microsoft Excel. Quantifications always represent the mean values of tested specimens. Analysis included error bars and the mean deviation. The total sample number is indicated in the corresponding figure legends or text sections. The different parameters were counted on captured images. Images were processed with Adobe Photoshop 7.0.

## Competing interests

The author(s) declare that they have no competing interests.

## Authors' contributions

AZ performed all the experiments and wrote the first draft of the manuscript. GG generated the Sp8 conditional knockout mice. AS and AM designed the experiments and wrote the final version of the manuscript. All authors approved the manuscript.

## Supplementary Material

Additional data file 1 *Sp8 *mRNA is expressed in Emx2KO and Pax6KO cortices. ISH using **(a-c, e) ***Sp8 *and **(d) ***Sp9 *riboprobes on E12.5 coronal sections. *Sp8 *expression in the medial and dorsal pallium of Emx2KO and Pax6KO specimens appears indistinguishable from controls (a-c). *Sp8 *and *Sp9 *are expressed mostly in non-overlapping domains of the telencephalon (d, e). *Sp9 *is detected at the pallial-subpallial border (d). **(f) **Scheme illustrating the generation of the floxed *Sp8 *allele. **(g) **Southern blot analysis revealed successfully recombined *Sp8 *floxed alleles and the deletion of the wild-type (wt) *Sp8 *allele. Restriction enzymes: H, *Hin*dIII; K, *Kpn*I; P, *Pac*I; R1, *Eco*RI; R5, *Eco*RV; S, *Spe*I; X, *Xba*I). Click here for file

Additional data file 2 Histological analysis of conditional Sp8 mutant forebrains. Nissl stained coronal sections at different developmental stages, as indicated. **(a-f) **Wild type (Wt), **(a' -f') **cKO, **(a'' -f'') **cKO no midline (most severe phenotype observed). At E12.5, conditional Sp8 mutant tissue sections revealed a significant size reduction of the telencephalon and the morphological absence of the septum at rostral levels (a', a''). Caudally, the basal ganglia appear as a single eminence (b', b'') when compared to controls (b). At E15.5, cKO forebrains lacked the septum (c, c'). Due to an almost complete disgenesis of the midline, the lateral ventricles appear as a single aqueduct space (compare (c) with the area indicated by the asterisk in (c'')). At more caudal levels, there is no obvious difference between Sp8cKO and Sp8cKO no midline. However, when compared to wild-type embryos, the internal capsule of conditional mutants showed aberrant fiber bundles extending towards the basal telencephalon (arrows in (d, d', d'')). At E18.5, further reduction of the forebrain size and a variable dysgenesis of the midline were observed (e, e', e''). *Sp8*-deficient embryos typically lack a discernable corpus callosum (CC); instead, unilaterally probst bundles (PB) were apparent (e, e', e''). Click here for file

Additional data file 3 No evidence for cell cycle alterations in Sp8 mutants. Immunohistochemistry for **(a, a', b, b') **Tuj and **(c, c') **Tuj/Pax6 antibodies on coronal sections. Tuj staining revealed no difference in the thickness of the PP/CP at E10.5 (a, a'), E12.5 (b, b'), and E15.5 (c, c'). Therefore, premature differentiation is not evident in cKO. Estimation of cell cycle parameters, using **(e, e', f) **BrdU, **(e, e', g) **phosphor-histone H3, and **(d, d', h) **BrdU/IdU staining on E12.5 sections. None of the tested cell cycle parameters were significantly abnormal in Sp8 conditional mutants. Click here for file
