# Supplementary figures and images for: Genetic interplay between the transcription factors Sp8 and Emx2 in the patterning of the forebrain
Source: Neural Dev. 2007 Apr 30;2:8. doi: 10.1186/1749-8104-2-8 (PMC1868949; doi:10.1186/1749-8104-2-8)

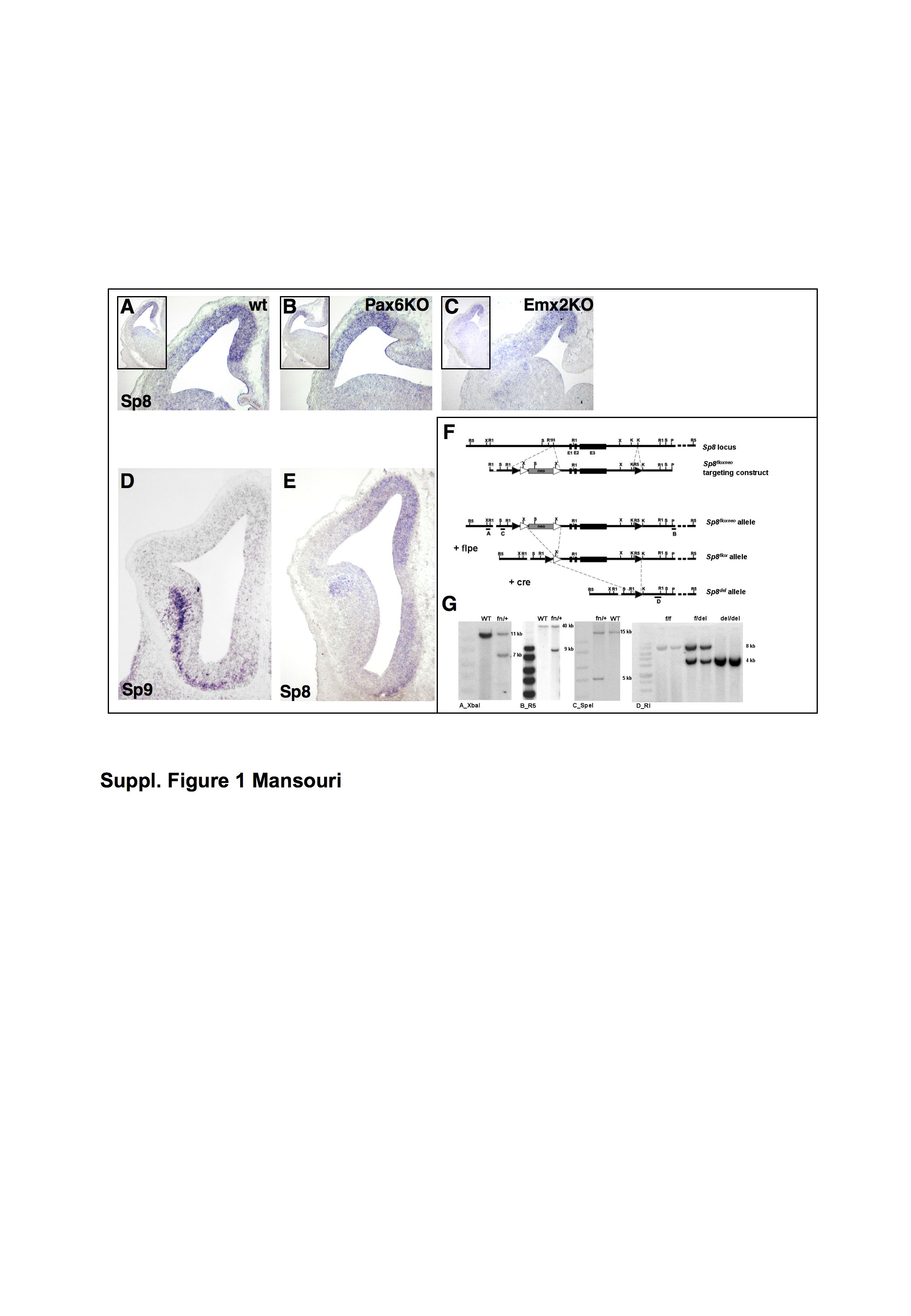

Supplement: Additional data file 1 — Sp8 mRNA is expressed in Emx2KO and Pax6KO cortices. ISH using (a-c, e) Sp8 and (d) Sp9 riboprobes on E12.5 coronal sections. Sp8 expression in the medial and dorsal pallium of Emx2KO and Pax6KO specimens appears indistinguishable from controls (a-c). Sp8 and Sp9 are expressed mostly in non-overlapping domains of the telencephalon (d, e). Sp9 is detected at the pallial-subpallial border (d). (f) Scheme illustrating the generation of the floxed Sp8 allele. (g) Southern blot analysis revealed successfully recombined Sp8 floxed alleles and the deletion of the wild-type (wt) Sp8 allele. Restriction enzymes: H, HindIII; K, KpnI; P, PacI; R1, EcoRI; R5, EcoRV; S, SpeI; X, XbaI). [file 1749-8104-2-8-S1.jpeg]

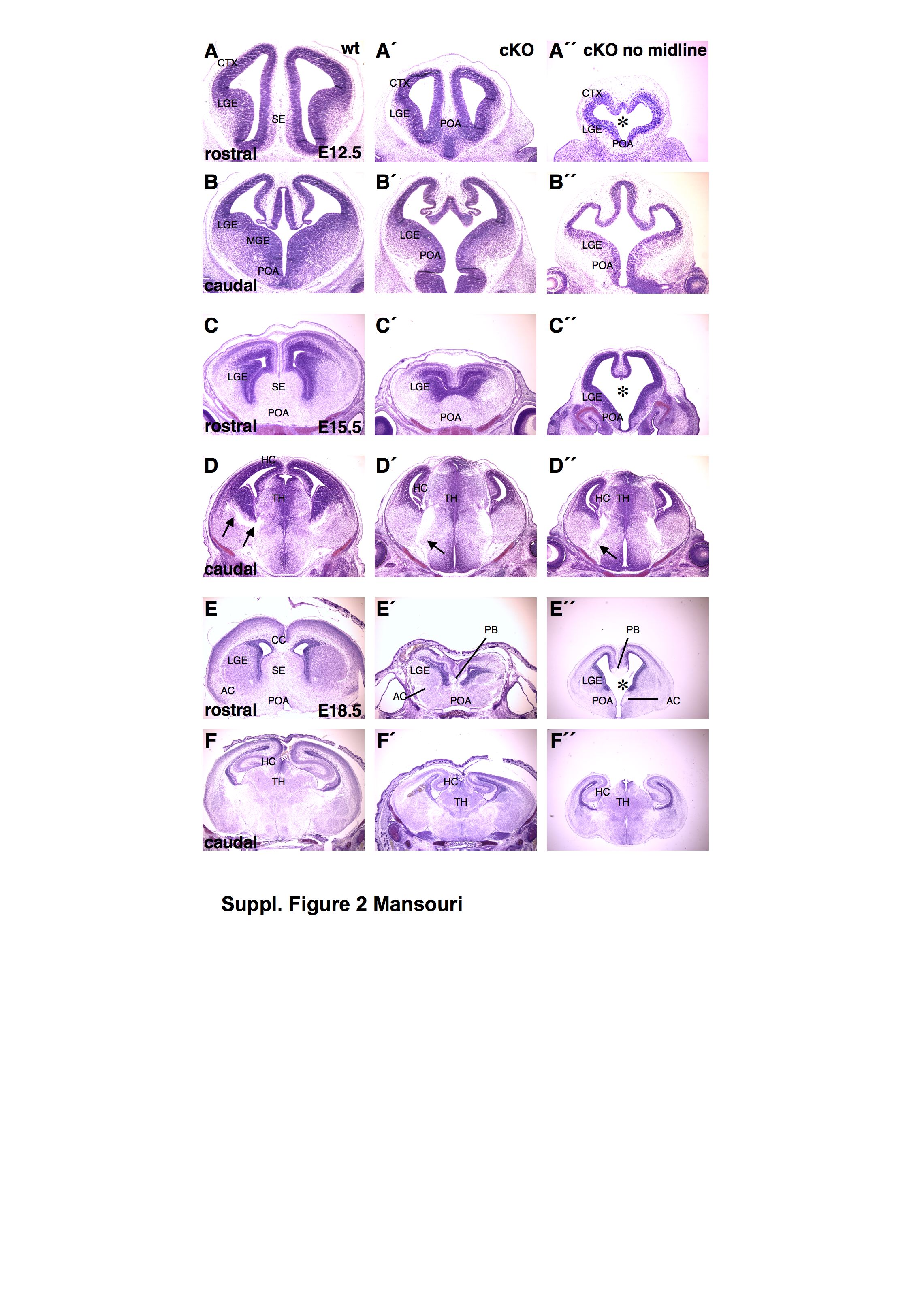

Supplement: Additional data file 2 — Histological analysis of conditional Sp8 mutant forebrains. Nissl stained coronal sections at different developmental stages, as indicated. (a-f) Wild type (Wt), (a' -f') cKO, (a'' -f'') cKO no midline (most severe phenotype observed). At E12.5, conditional Sp8 mutant tissue sections revealed a significant size reduction of the telencephalon and the morphological absence of the septum at rostral levels (a', a''). Caudally, the basal ganglia appear as a single eminence (b', b'') when compared to controls (b). At E15.5, cKO forebrains lacked the septum (c, c'). Due to an almost complete disgenesis of the midline, the lateral ventricles appear as a single aqueduct space (compare (c) with the area indicated by the asterisk in (c'')). At more caudal levels, there is no obvious difference between Sp8cKO and Sp8cKO no midline. However, when compared to wild-type embryos, the internal capsule of conditional mutants showed aberrant fiber bundles extending towards the basal telencephalon (arrows in (d, d', d'')). At E18.5, further reduction of the forebrain size and a variable dysgenesis of the midline were observed (e, e', e''). Sp8-deficient embryos typically lack a discernable corpus callosum (CC); instead, unilaterally probst bundles (PB) were apparent (e, e', e''). [file 1749-8104-2-8-S2.jpeg]

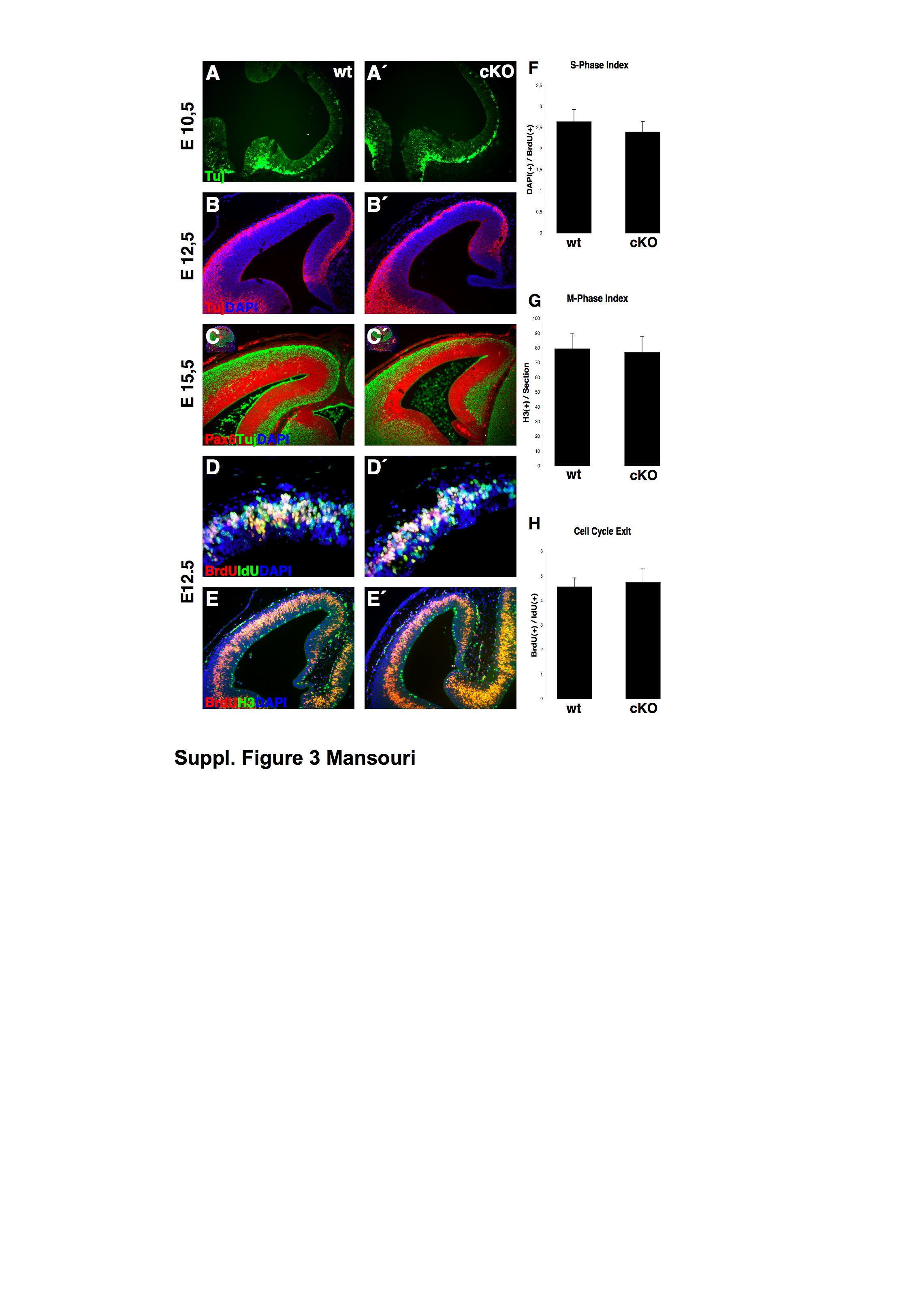

Supplement: Additional data file 3 — No evidence for cell cycle alterations in Sp8 mutants. Immunohistochemistry for (a, a', b, b') Tuj and (c, c') Tuj/Pax6 antibodies on coronal sections. Tuj staining revealed no difference in the thickness of the PP/CP at E10.5 (a, a'), E12.5 (b, b'), and E15.5 (c, c'). Therefore, premature differentiation is not evident in cKO. Estimation of cell cycle parameters, using (e, e', f) BrdU, (e, e', g) phosphor-histone H3, and (d, d', h) BrdU/IdU staining on E12.5 sections. None of the tested cell cycle parameters were significantly abnormal in Sp8 conditional mutants. [file 1749-8104-2-8-S3.jpeg]
